# Influence of long-term fertilization on soil aggregates stability and organic carbon occurrence characteristics in karst yellow soil of Southwest China

**DOI:** 10.3389/fpls.2023.1126150

**Published:** 2023-06-08

**Authors:** Yanling Liu, Meng Zhang, Han Xiong, Yu Li, Yarong Zhang, Xingcheng Huang, Yehua Yang, Huaqing Zhu, Taiming Jiang

**Affiliations:** ^1^ Institute of Soil and Fertilizer, Guizhou Academy of Agricultural Sciences, Guiyang, Guizhou, China; ^2^ Scientific Observing and Experimental Station of Arable Land Conservation and Agricultural Environment, Ministry of Agriculture, Guiyang, Guizhou, China

**Keywords:** long-term fertilization, yellow soil, aggregates stability, organic carbon, occurrence characteristics

## Abstract

Current research has long focused on soil organic carbon and soil aggregates stability. However, the effects of different long-term fertilization on the composition of yellow soil aggregates and the characteristics of the occurrence of organic carbon in the karst region of Southwest China are still unclear. Based on a 25-year long-term located experiment on yellow soil, soil samples from the 0–20 cm soil layer were collected and treated with different fertilizers (CK: unfertilized control; NPK: chemical fertilizer; 1/4 M + 3/4 NP: 25% chemical fertilizer replaced by 25% organic fertilizer; 1/2 M + 1/2 NP: 50% chemical fertilizer replaced by organic fertilizer; and M: organic fertilizer). In water-stable aggregates, soil aggregates stability, total organic carbon (TOC), easily oxidized organic carbon (EOC), carbon preservation capacity (CPC), and carbon pool management index (CPMI) were analyzed. The findings demonstrated that the order of the average weight diameter (MWD), geometric mean diameter (GWD), and macro-aggregate content (R_0.25_) of stable water aggregates was M > CK > 1/2M +1/2NP > 1/4M +3/4NP> NPK. The MWD, GWD, and R_0.25_ of NPK treatment significantly decreased by 32.6%, 43.2%, and 7.0 percentage points, respectively, compared to CK treatment. The order of TOC and EOC content in aggregates of different particle sizes was M > 1/2M +1/2NP > 1/4M +3/4NP> CK > NPK, and it increased as the rate of organic fertilizer increased. In macro-aggregates and bulk soil, the CPC of TOC (TOPC) and EOC (EOPC), as well as CPMI, were arranged as M > 1/2M +1/2NP > 1/4M +3/4NP> CK > NPK, but the opposite was true for micro-aggregates. In bulk soil treated with organic fertilizer, the TOPC, EOPC, and CPMI significantly increased by 27.4%–53.8%, 29.7%–78.1%, 29.7–82.2 percentage points, respectively, compared to NPK treatment. Redundancy analysis and stepwise regression analysis show that TOC was the main physical and chemical factor affecting the aggregates stability, and the TOPC in micro-aggregates has the most direct impact. In conclusion, the primary cause of the decrease in SOC caused by the long-term application of chemical fertilizer was the loss of organic carbon in macro-aggregates. An essential method to increase soil nutrient supply and improve yellow soil productivity was to apply an organic fertilizer to increase aggregates stability, storage and activity of SOC in macro-aggregates.

## 1 Introduction

Soil organic carbon (SOC) is an important indicator of soil fertility, which is crucial for increasing soil productivity, stability, and ability to reduce the global greenhouse effect (Luan et al., 2021). Soil aggregates are the fundamental components of soil structure, and the quantity and distribution of water-stable aggregates can provide insight into the stability and anti-erosion abilities of a soil structure (Ayoubi et al., 2020; Ma et al., 2022). Additionally, soil aggregates play a significant role in the transformation and accumulation of SOC (Six et al., 2004; Bidisha et al., 2010). Soil aggregates can protect SOC, a crucial cementing component required to form aggregates (Kamran et al., 2021). Therefore, aggregation and the sequestration of SOC are strongly correlated.

The application of fertilizer is a significant factor in the formation, evolution, and characterization of soil aggregates and a major factor in SOC pool fluctuations (Mustafa et al., 2021; Zhang et al., 2021a). Several studies have demonstrated that applying organic fertilizers can improve soil aggregates stability and increase the SOC content in aggregates (Abiven et al., 2009; Guo et al., 2019; Luan et al., 2019). Additionally, it has been discovered that high dosages of organic fertilizers and organic-inorganic mixes can reduce macro-aggregates’ stability and content (Zhang et al., 2020a). However, studies on the effects of chemical fertilizer application on soil aggregates and organic carbon content produced mixed results. According to some studies (Zhou et al., 2017), chemical fertilizer application decreased soil aggregates stability, but other studies claim that it increased soil aggregates stability and SOC stock (Adnan et al., 2020). Therefore, the findings addressing the effects of fertilizer treatment on the distribution and stability of soil aggregates and the distribution of SOC in aggregates vary according to the changes in soil types, climate, and crop (Possinger et al., 2020; Abiven et al., 2009).

Easily oxidized organic carbon (EOC) is an active organic carbon that may be directly used by soil organisms and affects the mineralization, migration, and degradation of exogenous carbon in the soil since it is the component of soil organic carbon that turns over most quickly (Ge et al., 2021). CPMI reflects the impact of external conditions on the changes in the quantity of EOC, and can comprehensively reflect the quality of SOC pool, the higher the CPMI, the higher the quality of SOC pool (Chaudhary et al., 2017; Ghosh et al., 2019). EOC and CPMI are more sensitive to changes in external environmental conditions such as land use transformation, fertilization management, agricultural field management than TOC (Wang et al., 2014). Therefore, understanding the EOC content and carbon pool management index (CPMI) variable characteristics in various particle size aggregates would help better understand SOC’s stability mechanism. However, prior studies on the sequestration of organic carbon in soil aggregates mostly concentrated on the content of TOC (Liu et al., 2019; Qiu et al., 2022), and there was comparatively little research on the active organic carbon in aggregates.

In the karst region of Southwest China, yellow soil is one of the most prevalent soil types—the main limiting factors to its high yield are poor soil structure and low fertility. Previous studies have shown that soil aggregates and organic carbon were closely related to the productivity of yellow soil (Zhang et al., 2016). Whereas the research on organic carbon mainly focuses on organic carbon in bulk soil (Zhang et al., 2021b), there is still insufficient research on soil aggregates stability, their carbon sequestration capacity, and SOC quality, the distribution of aggregates and their interrelationships with SOC content and quality under long-term fertilization are still unclear. Therefore, this study was based on a 25-year long-term location experiment of yellow soil to investigate the effects of long-term application of chemical and organic fertilizers on the composition and stability of soil aggregates, analyze the SOC and EOC contents in soil aggregates, and finally assess the carbon sequestration capacity (CPC) and CPMI. It also explored the relationship between soil aggregates stability, SOC content, and quality, which can provide a reference basis for structural improvement and SOC enhancement of yellow soil.

## 2 Materials and methods

### 2.1 Experimental site and materials

The experimental site was at the Guizhou Academy of Agricultural Sciences in Guiyang, Guizhou province (106°07’E, 26°11’N). The test site was located at an altitude of 1071 m, with an average annual temperature of 15.3°C, annual sunshine of 1354 hours, relative humidity of 75.5%, a frost-free period of 270 days, and annual precipitation is 1100–1200 mm. The yellow soil type at this site was classified as Acrisol in the World Reference Base for Soil Resources and was developed from the Triassic limestone and sand shale efflorescence. Prior to the long-term located experiment, which was started in 1995, the soil surface had the following physicochemical characteristics: a pH of 6.70, soil organic matter of 43.6 g·kg^-1^, total nitrogen of 2.05 g·kg^-1^, alkali-hydrolyzable nitrogen of 167.0 mg·kg^-1^, available phosphorus of 17.0 mg·kg^-1^, and available potassium of 109.0 mg·kg^-1^.

### 2.2 Experimental design and management

The five fertilization treatments selected for this study are as follows: CK treatment (no fertilizer), NPK treatment (chemical fertilizer), 1/4M +3/4NPtreatment (25% organic fertilizer replacing 25% chemical nitrogen and phosphorus fertilizer and all chemical potassium fertilizer), 1/2M +1/2NP treatment (50% organic fertilizer replacing 50% chemical nitrogen and phosphorus fertilizer and all chemical potassium fertilizer), and M treatment (organic fertilizer). All treatments, except CK treatment, used the same amounts of nitrogen fertilizer (**Table 1**). The fertilizers used in the experiment included urea (N, 46%), calcium superphosphate (P_2_O_5,_ 12%), and potassium chloride (K_2_O, 60%). Cow manure was the organic fertilizer used in the experiment; it contained 104.0 g·kg^-1^ of organic C, 2.7 g·kg^-1^ of N, 1.3 g·kg^-1^ of P_2_O_5_, and 6.0 g·kg^-1^ of K_2_O. Chemical nitrogen fertilizer was applied in two treatments during the seedling stage (40%) and the trumpet stage (60%), whereas phosphorus, potassium, and organic fertilizers were used as base fertilizers. The experiment used a maize monoculture that do not plant other crops after corn harvest. During the experiment, all agricultural activities were identical across all treatments except how fertilizer was applied.

**Table 1:** Nutrient application rates for various fertilization treatments.

Treatments	Chemical fertilizer (kg·hm^-2^)			Organic fertilizer (kg·hm^-2^)			Total nutrients (kg·hm^-2^)			Organic C (t·hm^-2^)
	N	P_2_O_5_	K_2_O	N	P_2_O_5_	K_2_O	N	P_2_O_5_	K_2_O	C
CK	0	0	0	0	0	0	0	0	0	0
NPK	165	82A.5	82.8	0	0	0	165	82.5	82.5	0
1/4M+3/4NP	123.8	62.7	0	41.2	19.8	91.6	165	82.5	91.6	1.58
1/2M+1/2NP	82.5	42.8	0	82.5	39.7	183.3	165	82.5	183.3	3.18
M	0	0	0	165	79.4	366.7	165	79.4	366.7	6.35

CK: unfertilized control; NPK: chemical fertilizer; 1/4M+3/4 NP: 25% chemical fertilizer replaced by 25% organic fertilizer; 1/2M+1/2NP: 50% chemical fertilizer replaced by 50% organic fertilizer; M: organic fertilizer.

### 2.3 Sampling and measurement

After the maize harvest in September 2019, soil samples were collected using a five-point sampling method from the 0–20 cm soil layer. Soil disturbance was minimized during soil sample collection to preserve the integrity of the soil structure. The soil samples were taken back to the laboratory, stripped of their roots, cut into small pieces along the natural structure, and finally air-dried for use. Bao’s method determined the soil’s chemical properties (Bao, 2000). A 1:2.5 extraction mixture (soil/water, w/v) was used to determine the soil’s pH using a pH meter (FE20K, Mettler Toledo, Zurich, Switzerland). The potassium dichromate-external heating method determined the soil’s total organic carbon (TOC). The Kjeldahl method was used for the total N. The ring knife method calculates the bulk density. The laser particle size analyzer (MS3000, Britain) was used to determine the clay content. The EOC content was determined using 333 mmol·L^-1^ of the potassium permanganate oxidation method (Ge et al., 2021).

According to Li et al.’s (2020) method, soil mechanical-stable aggregates (MSAs) were performed. A vibrating sieve (GRINDER SS200) with an amplitude of 2.0 mm and a sieving time of 10 min was used to sieve a 400 g mixed soil sample with pore sizes of 5, 2, 1, 0.5, and 0.25 mm—the weight of the soil after sieving was determined for each pore size.

The soil’s water-stable aggregates (WSAs) were identified using the wet-sieving method (Hong et al., 2021). In the water-stable aggregate instrument (Daiki DIK-2012), a 50 g air-dried soil sample was placed on top of the sieve set (5, 2, 1, 0.5, and 0.25 mm successively). The bucket was gradually filled with distilled water until the top of the sieve was submerged. It took 10 min of oscillation at a frequency of 30 times per minute and an amplitude of 40 mm to separate the soil WSAs. After carefully removing the sieves, the soil in each sieve was transferred into the aluminum box using small water streams. Calculations were done to determine the percentage content of the WSAs of various particle sizes after the separated samples were dried at 80°C and weighed.

### 2.4 Calculation formula

The dry sieving method’s *R*_0.25_ content and the wet sieving method’s *R*_0.25_ content were expressed as DR_0.25_ and WR_0.25_, respectively (Sun et al., 2021). *R*_0.25_, percentage of aggregate destruction (PAD), unstable aggregate index (E_LT_), mean weight diameter (MWD), and geometric mean diameter (GWD) was calculated as follows:


(1) $$R_{0.25}={\text{Mr}}_{>0.25}/{\text{M}}_{\text{t}}$$

(2) $$\text{PAD}=\frac{\left({\text{DR}}_{0.25}-{\text{WR}}_{0.25}\right)}{{\text{DR}}_{0.25}}\times 100\%$$

(3) $${\text{E}}_{\text{LT}}=\frac{\left({\text{M}}_{\text{t}}-{\text{WR}}_{0.25}\right)}{{\text{M}}_{\text{t}}}\times 100\%$$

(4) $$\text{MWD}=\sum \left({\text{x}}_{\text{i}}\times \frac{{\text{M}}_{\text{i}}}{{\text{M}}_{\text{t}}}\right)$$

(5) $$\text{GMD}={\text{E}}_{\text{xp}}\left[\frac{\left({\text{M}}_{\text{i}}\times \ln{\text{X}}_{\text{i}}\right)}{{\text{M}}_{\text{i}}}\right]$$

where, *R*_0.25_ denoted the content of aggregates bigger than 0.25 mm. Mt denoted the total weight of aggregates. Mr_>0.25_ denoted the weight of aggregates with particle size larger than 0.25 mm. X_i_ denoted mean diameter of soil aggregates (mm) and M_i_ denoted the weight of soil aggregates of each class (>5 mm, 2-5 mm, 1-2 mm, 0.5-1 mm, 0.25-0.5 mm).

According to Dixit et al. (2020), soil aggregate carbon sequestration capacity (CPC) and organic carbon pool management index (CPMI) were calculated. CPC, carbon pool index (CPI), carbon pool activity (CPA), carbon pool activity index (CPAI), and CPMI were calculated as follows:


(6) Hardly oxidized organic carbon(HOC)=TOC–EOC

(7) $${\text{CPC}}_{\text{i}}={\text{SSAC}}_{\text{i}}\times {\text{W}}_{\text{i}}$$

(8) $${\text{CPC}}_{\text{bulk soil}}={\text{CPC}}_{\text{macro-aggregates}}+{\text{CPC}}_{\text{micro-aggregates}}$$

where CPC_i_ denoted the carbon preservation capacity of Level i soil aggregates. SSAC_i_ denoted the content of organic carbon (TOC, EOC, and HOC) in soil. W_i_ denoted the percentage of soil aggregates of each class (>5 mm, 2-5 mm, 1-2 mm, 0.5-1 mm, 0.25-0.5 mm). CPC _bulk soil_ denoted CPC in bulk soil, CPC _macro-aggregates_ denoted CPC in macro-aggregates, CPC _micro-aggregates_ denoted CPC in micro-aggregates.


(9) CPI=(TOC in sample soil)/(TOC in reference soil)

(10) CPA=EOC/NOC

(11) CPAI=(CPA of soil sample)/(CPA of reference soil)

(12) CPMI=CPI×CPAI×100

In this study, the CK treatment soil was used as a reference soil for the calculation.

### 2.5 Statistical analysis

Excel 2010 was used to calculate the experimental data. The Canoco for Windows 4.5 program performed redundancy analysis (RDA). Statistical Package for Social Sciences was used for variance, correlation, and path analyses. One-way analysis of variance and Duncan’s multiple range test (*p<* 0.05) were used to examine the differences between treatments.

## 3 Results

### 3.1 Soil physicochemical properties

NPK treatment significantly decreased soil organic matter by 15.5% compared to CK treatment (**Table 2**). Conversely, treatments with organic fertilizer significantly increased pH and organic matter by 7.5%–13.5% and 28.9%–52.1%, significantly decreased soil bulk density and clay content by 3.3%–10.0% and 1.7–3.7 percentage points, respectively.

**Table 2:** Soil physicochemical properties under long-term different fertilization treatments.

Treatments	pH	Organic matter/OM (g·kg^-1^)	Bulk density/BD (g·cm^-3^)	Clay content/CC (%)
CK	6.50 ± 0.01 c	48.4 ± 0.33 c	1.23 ± 0.01 a	20.0 ± 1.60 a
NPK	6.50 ± 0.20 c	40.9 ± 2.56 d	1.20 ± 0.03 a	19.7 ± 0.51 a
1/4M+3/4NP	6.99 ± 0.10 b	56.3 ± 2.14 b	1.15 ± 0.02 b	18.0 ± 0.89 b
1/2M+1/2NP	7.25 ± 0.03 a	52.7 ± 3.56 b	1.16 ± 0.02 b	17.4 ± 0.68 b
M	7.38 ± 0.01 a	62.2 ± 3.65 a	1.08 ± 0.03 c	16.0 ± 1.82 c

Different lowercase letters in the same column indicated significant difference at p<0.05.

### 3.2 Composition and soil aggregates stability

**Figure 1:**
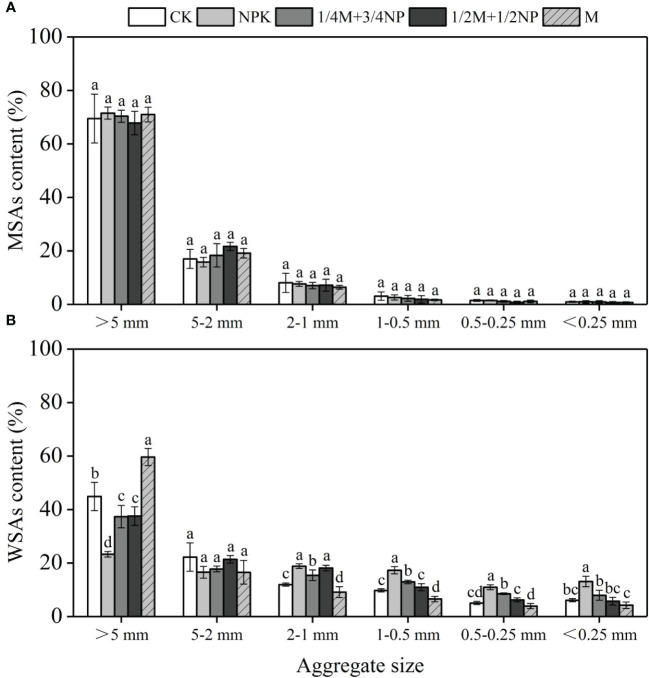
Effect of long-term fertilization on the distribution of mechanical-stable aggregates (MSAs, **A**) water-stable aggregate (WSAs, **B**). Different lowercase letters indicate significant difference at *p*<0.05.

**Figure 1** shows how MSAs and WSAs are distributed in yellow soil (**Figure 1A**). The proportion of aggregates gradually decreased with decreasing particle size in both MSAs and WSAs, which were predominate>5 mm aggregates. Interestingly, long-term fertilization had little effect on MSAs (**Figure 1A**) but significantly changed the distribution of WSAs (**Figure 1B**). In WSAs, the content of >5 mm aggregates of NPK treatment was significantly decreased by 21.6 percentage points compared to the CK treatment. In contrast, the content of 2–0.25 mm aggregates was significantly increased by 5.9–7.5 percentage points, and the content of<0.25 mm micro-aggregates was increased by 7.0 percentage points. Treatments with organic fertilizer significantly increased the content of >5 mm aggregates by 14.1–36.4 percentage points compared to the NPK treatment, but they significantly decreased the content of 2–0.25 mm aggregates by 0.7–10.8 percentage points. The content of<0.25 mm micro-aggregates also significantly decreased by 5.2–8.9 percentage points.

Compared to CK treatment, the PAD and E_LT_ of NPK treatment significantly increased by 6.98 percentage points and 7.01 percentage points, respectively, whereas the MWD, GWD, and W*R*_0.25_ significantly decreased by 32.6%, 43.2%, and 7.0 percentage points, respectively (**Table 3**). The MWD and GWD with 1/4M +3/4NPand 1/2M +1/2NP treatments were significantly decreased when compared to CK treatment, whereas M treatment significantly increased them by 13.9% and 22.2%, respectively. Compared to NPK treatment, organic fertilizer treatments significantly decreased PAD and E_LT_ by 5.11–8.66 percentage points and 5.16–8.87 percentage points, respectively, whereas significantly increasing MWD, GWD, and W*R*_0.25_ by 28.7%–69.1%, 39.9%–115.2%, and 5.2–86.9 percentage points. This indicated that long-term chemical fertilizers could decrease soil aggregates stability, whereas long-term application of organic fertilizers could increase soil aggregates stability.

**Table 3:** Soil aggregates stability under long-term different fertilization treatments.

Treatments	Soil aggregate		Soil water stable aggregates		
	PAD (%)	E_LT_ (%)	MWD (mm)	GMD (mm)	WR0.25 (%)
CK	5.22 ± 0.57 bc	6.09 ± 0.57 bc	3.31 ± 0.08 b	2.43 ± 0.04 b	93.9 ± 1.52 ab
NPK	12.2 ± 1.92 a	13.1 ± 1.93 a	2.23 ± 0.07 d	1.38 ± 0.07 e	86.9 ± 1.58 c
1/4M+3/4NP	7.09 ± 2.08 b	7.94 ± 1.87 b	2.87 ± 0.17 c	1.93 ± 0.17 d	92.1 ± 1.52 b
1/2M+3/2NP	5.10 ± 1.50 bc	5.73 ± 1.43 bc	3.02 ± 0.10 c	2.17 ± 0.09 c	94.3 ± 1.17 ab
M	3.54 ± 0.99 c	4.23 ± 1.20 c	3.77 ± 0.02 a	2.97 ± 0.12 a	95.8 ± 0.98 a

Different lowercase letters in the same column indicated significant difference at p<0.05.

### 3.3 Soil aggregate-associated TOC and EOC contents

**Figure 2:**
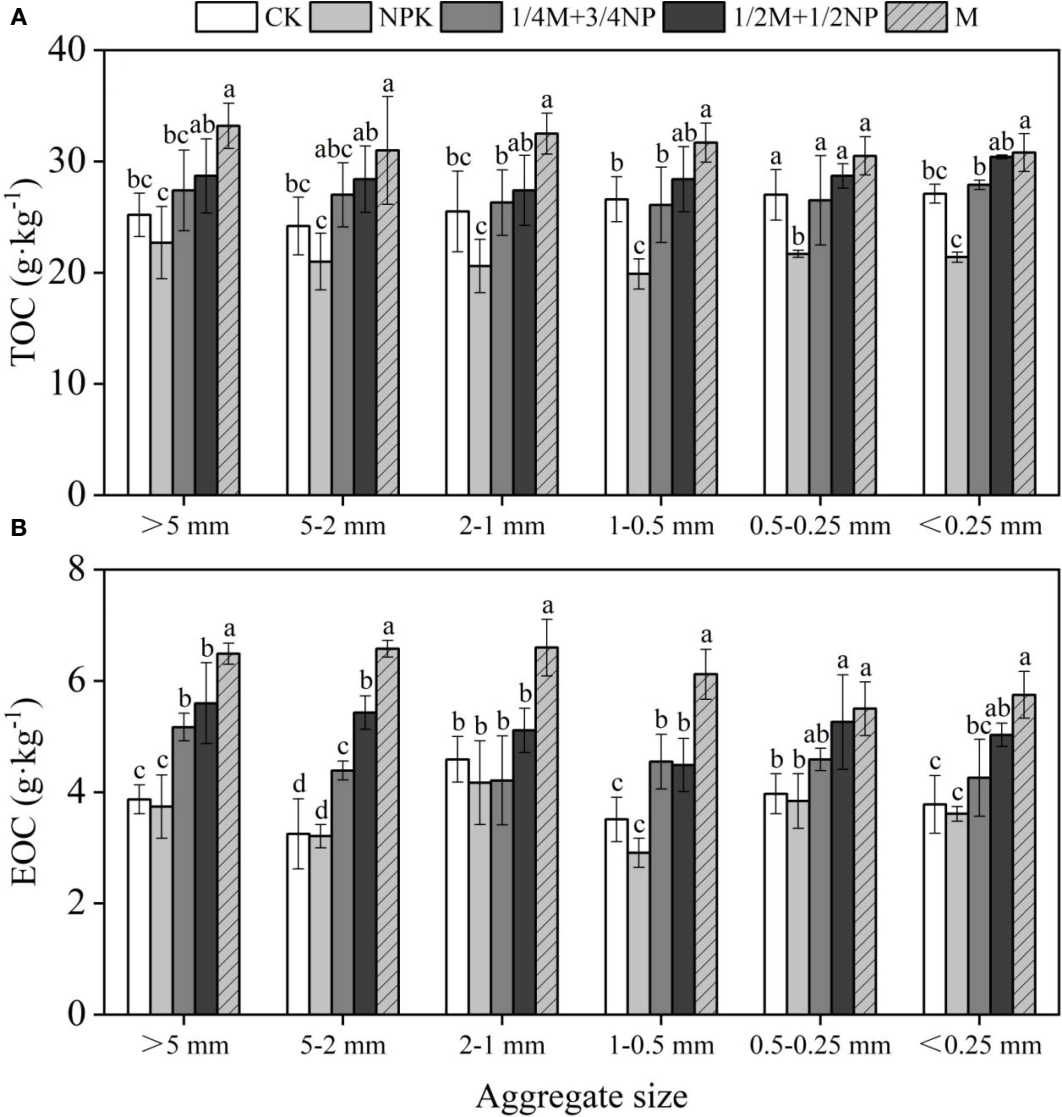
Effect of long-term fertilization on the content of TOC **(A)** and EOC **(B)** in yellow soil. Different lowercase letters indicate significant difference at *p*<0.05.

In aggregates of various particle sizes, the contents of TOC (**Figure 2A**) and EOC (**Figure 2B**) were grouped in the following order: M > 1/2M +1/2NP > 1/4M +3/4NP> CK > NPK, which increased with the increase in organic fertilizer rates. The TOC content of the organic fertilizer treatments increased by 1.32%–31.2% in different particle sizes compared to the NPK treatment. The EOC content of the organic fertilizer treatments increased by 0.96%–56.4% in different particle sizes compared to the NPK treatment.

### 3.4 Water-stable aggregate-associated TOC stock

Long-term fertilization applications significantly affected the TOPC in soil water-stable aggregates (**Table 4**). When compared to CK treatment, the TOPC of NPK treatment was significantly decreased by 22.4% in macro-aggregates (>0.25 mm), whereas TOPC was significantly increased by 69.7% in micro-aggregates (<0.25 mm). Moreover, compared to NPK treatment, the TOPC of treatments with organic fertilizer was significantly increased by 34.8%–69.6% in macro-aggregates but significantly decreased by 22.1%–50.4% in micro-aggregates. Additionally, the fluctuation pattern of TOPC in bulk soil was similar to that of macro-aggregates.

**Table 4:** Soil aggregate-associated total organic carbon preservation capacity under long-term different fertilization treatments.

Treatments	Aggregate-associated TOPC (g·kg^-1^)							TOPC in bulk soil (g·kg^-1^)
	>5 mm	5-2 mm	2-1 mm	1-0.5 mm	0.5-0.25 mm	<0.25 mm	>0.25 mm	
CK	11.2 ± 0.56 b	5.45 ± 1.85 a	3.06 ± 0.56 b	2.60 ± 0.05 bc	1.35 ± 0.06 cd	1.65 ± 0.25 bc	23.7 ± 1.90 b	25.4 ± 2.14 bc
NPK	5.26 ± 0.55 c	3.48 ± 0.68 a	3.86 ± 0.30 ab	3.44 ± 0.40 a	2.38 ± 0.19 a	2.80 ± 0.41 a	18.4 ± 1.81 c	21.2 ± 1.57 c
1/4M+3/4NP	10.3 ± 2.17 b	4.82 ± 0.77 a	4.03 ± 0.41 ab	3.38 ± 0.53 ab	2.27 ± 0.41 ab	2.19 ± 0.35 b	24.8 ± 3.36 b	27.0 ± 3.22 b
1/2M+1/2NP	10.7 ± 0.28 b	6.10 ± 1.03 a	4.98 ± 0.87 a	3.14 ± 0.67 ab	1.79 ± 0.27 bc	1.72 ± 0.30 bc	26.7 ± 2.67 ab	28.5 ± 2.60 ab
M	19.8 ± 0.46 a	5.26 ± 2.24 a	2.98 ± 0.71 b	2.06 ± 0.24 c	1.19 ± 0.30 d	1.39 ± 0.27 c	31.2 ± 2.56 a	32.6 ± 2.31 a

TOPC represent capacity the CPC of TOC. Different lowercase letters in the same column indicate significant difference (p<0.05).

**Figure 3:**
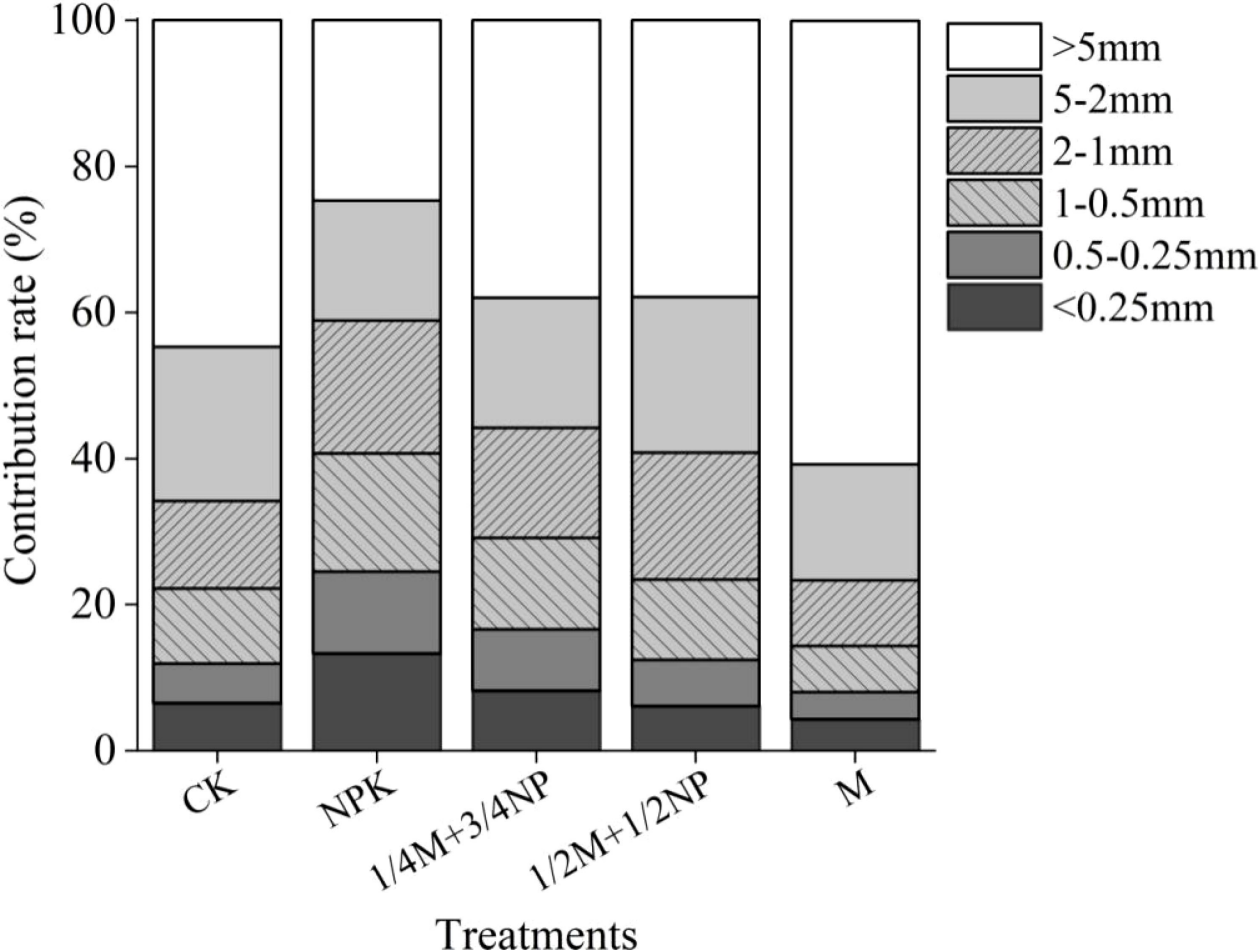
Contribution rate of aggregate-associated TOC to TOC in bulk soil under long-term different fertilization treatments.

The TOC contribution rate (TOCR) in aggregates >5 mm was the highest (24.7%–60.7%) among all treatments (**Figure 3**). M treatment has the highest SOCR in aggregates >5 mm, NPK treatment has the lowest, and vice versa in aggregates<2 mm. NPK treatment significantly increased the TOCR in micro-aggregates by 6.78% compared to CK treatment. On the other hand, the TOCR in micro-aggregates of treatments with organic fertilizer significantly decreased by 5.07%–8.97% compared to NPK treatment, with the decline increasing with the rate of organic fertilizer.

### 3.5 Carbon pool composition of water-stable aggregates

Compared to the NPK treatment, organic fertilizer treatments significantly increased the CPC of EOC (EOPC) by 39.1%–97.8% in macro-aggregates and 29.7%–78.1% in bulk soil, but decreased by 29.8%–48.9% in micro-aggregates (**Table 5**). Similarly, the CPC of HOC (HOPC) of treatments with organic fertilizer significantly increased by 34.0%–62.1% in macro-aggregates and 26.7%–47.7% in bulk soil, but decreased by 20.6%–50.6% in micro-aggregates. HOC comprised the majority of TOC, and the HOC/TOC ratio was >80%. For the M treatment, macro-aggregates, micro-aggregates, and bulk soil had the highest EOC/TOC, significantly increased by 3.1–4.7 percentage points compared to CK treatment.

**Table 5:** Changes of soil organic carbon pool composition in aggregates under long-term different fertilization treatments.

Aggregate particle size	Treatments	EOPC (g·kg^-1^)	HOPC (g·kg^-1^)	EOC/TOC (%)	HOC/TOC (%)
Macro-aggregate	CK	3.57 ± 0.25 d	20.1 ± 1.65 b	15.1 ± 0.18 b	84.9 ± 0.18 a
	NPK	3.12 ± 0.15 d	15.3 ± 1.96 c	17.1 ± 2.59 ab	82.9 ± 2.59 ab
	1/4M+3/4NP	4.34 ± 0.33 c	20.5 ± 3.04 b	17.6 ± 1.16 ab	82.4 ± 1.16 ab
	1/2M+1/2NP	5.00 ± 0.37 b	21.7 ± 2.31 ab	18.7 ± 0.53 a	81.3 ± 0.53 b
	M	6.17 ± 0.20 a	24.8 ± 2.41 a	19.8 ± 1.17 a	80.2 ± 1.17 b
Micro-aggregate	CK	0.23 ± 0.04 b	1.42 ± 0.25 bc	14.1 ± 2.81 b	85.9 ± 2.81 a
	NPK	0.47 ± 0.07 a	2.33 ± 0.35 a	16.9 ± 0.81 ab	83.1 ± 0.81 b
	1/4M+3/4NP	0.33 ± 0.05 b	1.85 ± 0.31 ab	15.2 ± 1.20 ab	84.8 ± 1.20 ab
	1/2M+1/2NP	0.26 ± 0.06 b	1.43 ± 0.24 bc	16.6 ± 0.66 ab	83.4 ± 0.66 ab
	M	0.24 ± 0.05 b	1.15 ± 0.22 c	17.2 ± 0.51 a	82.8 ± 0.51 b
Bulk soil	CK	3.80 ± 0.25 d	21.6 ± 1.90 bc	15.0 ± 0.36 c	85.0 ± 0.36 a
	NPK	3.60 ± 0.18 d	17.6 ± 1.74 c	17.1 ± 2.13 bc	82.9 ± 2.13 ab
	1/4M+3/4NP	4.67 ± 0.32 c	22.3 ± 2.90 ab	17.4 ± 0.95 b	82.6 ± 0.95 b
	1/2M+1/2NP	5.29 ± 0.37 b	23.2 ± 2.24 ab	18.6 ± 0.49 ab	81.4 ± 0.49 bc
	M	6.41 ± 0.16 a	26.0 ± 2.22 a	19.7 ± 1.12 a	80.0 ± 1.12 c

EOPC represents the CPC of EOC. HOPC represents the CPC of HOC, EOC represents easily oxidized organic carbon. HOC represents hardly oxidized organic carbon. Different lowercase letters in the same column indicated significant difference at p<0.05.

### 3.6 Soil CPMI of water-stable aggregates

CPI of M treatment significantly increased by 70.5% in macro-aggregates and 54.8% in bulk soil, but it decreased by 50.3% in micro-aggregates compared to NPK treatment (**Table 6**). The M treatment had the highest CPA and CPAI of all the treatments, but the CK treatment had the lowest CPA and CPAI. In macro-aggregates and bulk soil, the CPMI was arranged in the following order: M> 1/2M +1/2NP > 1/4M +3/4NP> CK > NPK, but the opposite in micro-aggregates. The CPMI of the organic fertilizer treatment was significantly increased than that of the NPK treatment by 40.1%–104.8% in macro-aggregates and 30.6%–84.5% in bulk soil, but it was significantly decreased by 32.7%–48.0% in micro-aggregates.

**Table 6:** Soil carbon pool management index of aggregates with different particle sizes under long-term different fertilization treatments.

Aggregate particle size	Treatments	CPI	CPA	CPAI	CPMI
Macro-aggregate	CK	1.00 ± 0.00 bc	0.18 ± 0.00 b	1.00 ± 0.00 b	100.0 ± 0.0 c
	NPK	0.78 ± 0.02 c	0.21 ± 0.04 a	1.17 ± 0.19 ab	89.9 ± 11.5 c
	1/4M+3/4NP	1.06 ± 0.14 abc	0.21 ± 0.02 ab	1.21 ± 0.12 ab	126.0 ± 6.7 b
	1/2M+1/2NP	1.13 ± 0.23 ab	0.23 ± 0.01 a	1.30 ± 0.09 a	147.0 ± 19.7 b
	M	1.33 ± 0.24 a	0.25 ± 0.02 a	1.40 ± 0.16 a	184.1 ± 12.1 a
Micro-aggregate	CK	1.00 ± 0.00 c	0.16 ± 0.05 b	1.00 ± 0.00 a	100.0 ± 0.0 b
	NPK	1.69 ± 0.09 a	0.20 ± 0.01 a	1.29 ± 0.52 a	217.3 ± 77.0 a
	1/4M+3/4NP	1.33 ± 0.29 b	0.18 ± 0.01 ab	1.12 ± 0.28 a	146.2 ± 3.5 b
	1/2M+1/2NP	1.04 ± 0.04 c	0.20 ± 0.00 ab	1.26 ± 0.46 a	131.7 ± 53.6 b
	M	0.84 ± 0.13 c	0.21 ± 0.01 a	1.32 ± 0.50 a	112.9 ± 58.4 b
Bulk soil	CK	1.00 ± 0.00 bc	0.18 ± 0.01 b	1.00 ± 0.00 c	100.0 ± 0.0 d
	NPK	0.84 ± 0.01 c	0.23 ± 0.03 a	1.17 ± 0.12 bc	97.3 ± 8.2 d
	1/4M+3/4NP	1.08 ± 0.15 abc	0.22 ± 0.01 ab	1.20 ± 0.13 abc	127.1 ± 6.5 c
	1/2M+1/2NP	1.13 ± 0.21 ab	0.24 ± 0.01 a	1.30 ± 0.11 ab	145.7 ± 15.2 b
	M	1.30 ± 0.21 a	0.25 ± 0.02 a	1.39 ± 0.18 a	179.5 ± 6.8 a

Different lowercase letters in the same column indicate significant differences at p<0.05 in different treatments of the same aggregate particle size.

### 3.7 Relationship between aggregates stability and organic carbon

**Figure 4:**
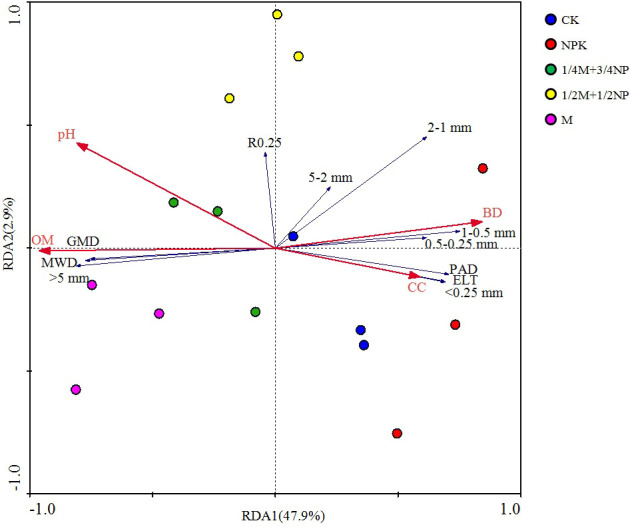
Redundancy analysis of the soil physicochemicals indexes and the composition and stability of soil aggregate.

The redundancy analysis findings revealed significant differences among various treatments in soil aggregates’ composition and stability characteristics. Similarities between CK and NPK, 1/2M +1/2NP, 1/4 M + 3/4 NP, and M were found (**Figure 4**). The physicochemical properties of the soil could explain the composition and stability of water-stable aggregates. The overall explanation rate was 51.4%, with RDA1 accounting for 47.9% and RDA2 accounting for 2.9%. The main environmental factor influencing the composition and stability of water-stable aggregates was organic matter (OM). OM was positively correlated with the content of >5 mm aggregates, GMD, and WMD and negatively correlated with the content of<2 mm all particle size aggregates, PAD, and E_LT_.

**Figure 5:**
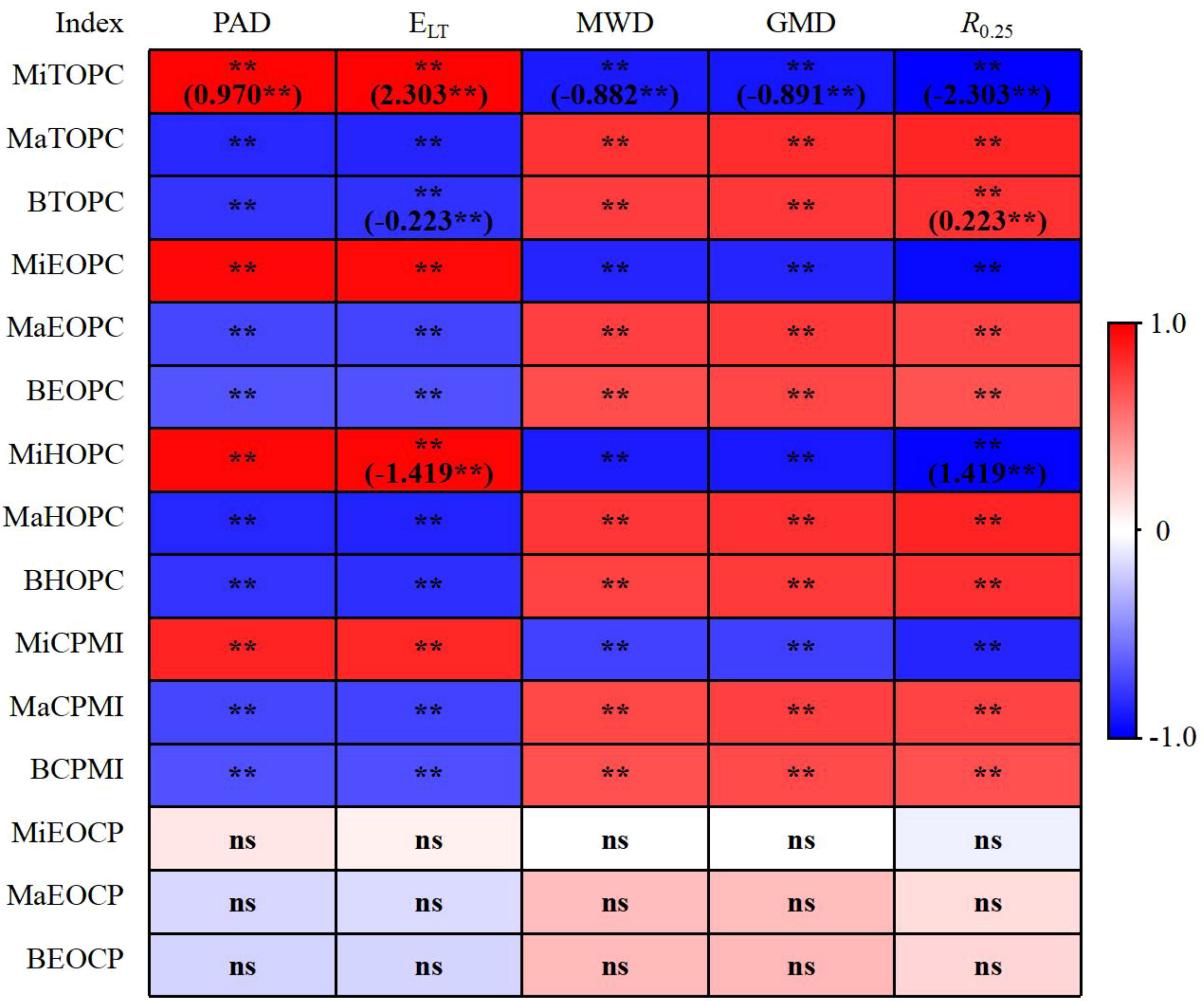
Correlation coefficient and path coefficient between soil aggregate stability and soil organic carbon pool. MiTOPC represents TOPC in micro-aggregate. MaTOPC represents TOPC in macro-aggregate. BTOPC represents TOPC in bulk soil. MiEOPC represents EOPC in micro-aggregate. MaEOPC represents EOPC in macro-aggregate. BEOPC represents EOPC in bulk soil. MiHOPC represents HOPC in micro-aggregate. MaHOPC represents HOPC in macro-aggregate. BHOPC represents HOPC in bulk soil. MiCPMI represents CPMI in micro-aggregate. MaCPMI represents CPMI in macro-aggregate. BCPMI represents CPMI in bulk soil. MiEOCP represents EOC/TOC in micro-aggregate. MaEOCP represents EOC/TOC in macro-aggregate. BEOCP represents EOC/TOC in bulk soil. ** represents significant at *p*<0.01; ns, no significant. The values in parentheses represent the path coefficient.

Soil aggregates stability and the organic carbon associated with aggregates were analyzed for association (**Figure 5**). The findings demonstrated that TOPC, EOPC, HOPC, and CMPI in the aggregates significantly or highly significantly correlated with soil aggregates stability. Moreover, soil aggregates stability positively correlated with CPC in macro-aggregates and bulk soil but negatively correlated with CPC in micro-aggregates. Additionally, MiTOPC also had the largest direct path coefficient for the soil aggregates stability index, which suggested that it had a significant direct impact on soil aggregates stability, according to the results of the stepwise regression analysis.

## 4 Discussion

### 4.1 Effects of long-term fertilization on the composition and soil aggregates stability

According to aggregate hierarchical theory, aggregates can be classified as macro-aggregates (>0.25 mm), micro-aggregates (<0.25–0.053 mm), and silt-clay particles (<0.053 mm) (Oades and Waters, 1991). Larger (>2 mm) and smaller (2–0.25 mm) macro-aggregates make up macro-aggregates (Brown et al., 2014). The stability of soil aggregates was decreased in this study due to the long-term application of chemical fertilizer, which caused >5 mm larger macro-aggregates to decompose into smaller macro-aggregates and micro-aggregates. The reason may be related to the significant decrease in soil organic matter content and the formation of organic binders following long-term chemical fertilizers application (Yu et al., 2012). However, the long-term application of organic fertilizers may promote the transformation of smaller macro-aggregates and micro-aggregates into >5 mm larger macro-aggregates, improving the stability of soil aggregates. For this reason, organic fertilizer applications could provide the soil with exogenous organic matter, increase soil microbial activity and plant organic residues, and provide the cementing material required for soil aggregates formation (Mustafa et al., 2021; Huang et al., 2022).

### 4.2 Effects of long-term fertilization on the composition of organic carbon pool in yellow soil aggregates

It has been extensively discovered that soil carbon conservation capacity and carbon inputs are positively correlated (Zhang et al., 2010). However, some studies have shown that the soil TOC content does not significantly increase with high carbon inputs, and there is a phenomenon of carbon saturation (Zhang et al., 2012). Results of other studies have shown that the TOC content was positively correlated with carbon input in macro-aggregates and bulk soil. In contrast, the TOC content was not insensitive to carbon input in micro-aggregate (Di et al., 2021). This study found that SOC had not yet reached saturation because the TOC content increased as the rate of organic fertilizer was increased in aggregates of different particle sizes and bulk soil. This indicates that the carbon sequestration potential of yellow soil was high.

EOC and CPMI can more accurately reflect changes in soil fertility and the transformation of the SOC pool. This study showed that long-term application of organic fertilizer could significantly increase EOPC, EOC/TOC, and CPMI in bulk soil. The reason could be that long-term application of organic fertilizer improves soil fertility, promotes the growth of above-ground plants, and increases the return of organic plant residues, which further promotes the accumulation of EOC and improves the quality of soil carbon pool (Chen et al., 2019; Huang et al., 2019; Tian et al., 2021). Applying chemical fertilizers improves the return of plant organic residues by increasing plant biomass but also consumes active organic carbon in the soil for crop growth. Therefore, there is no significant impact on EOC compare to CK treatment (Sarker et al., 2018). According to some studies, the SOC pool is more unstable the higher the soil EOC, which decreases TOC (Yuan et al., 2021). In this study, there was a significant positive correlation between TOC and EOC, possibly due to the increase in both EOC and HOC caused by exogenous organic carbon input. This indicates that the application of organic fertilizers could improve the activity of SOC pool, and does not reduce its stability.

The contribution rate of macro-aggregates combined organic carbon to TOC in this study, under various fertilization modes, was >86.7%, indicating that macro-aggregates combined organic carbon was the predominant component of SOC. So the macro-aggregates associated with organic carbon were the predominant loss type when long-term application of chemical fertilizer, whereas it’s the predominant storage type when long-term application of organic fertilizer. According to the theory of multistage aggregation (larger aggregates are composed of smaller aggregates and organic cementitious substances), the SOC sequestration law in macro-aggregates of each treatment was consistent with the SOC sequestration law in bulk soil (Huang et al., 2022). Therefore, long-term chemical fertilizer application significantly increased micro-aggregate content, improving the TOPC in micro-aggregates and their contribution to TOC in bulk soil. The reason was that micro-aggregates have a higher specific surface area, which could increase aggregates’ CPC (Zhang et al., 2020b). On the other hand, insufficient external organic carbon supply might promote the decomposition of SOC, which would then trigger the first decomposition of macro-aggregates combined organic carbon, lowering the TOPC in those macro-aggregates (Mustafa et al., 2021). Therefore, long-term application of organic fertilizer significantly improved the TOPC in macro-aggregates and its contribution rate to TOC in bulk soil because exogenous organic matter increased aggregate organic cement and promoted the transformation of small aggregates to large aggregates (Karami et al., 2012).

### 4.3 Relationship between soil aggregates stability and organic carbon pool

Agglomeration, which can protect organic carbon, and the preservation of soil organic carbon were closely associated, whereas the presence of organic carbon promoted the formation and stability of aggregates (Xu and Wang, 2017; Cao et al., 2021). According to the RDA findings in this study, the amount of soil organic matter (OM) had the greatest impact on the soil aggregates stability. Meanwhile, the stability of soil aggregates was positively correlated with CPC in macro-aggregates and bulk soil and negatively correlated with CPC in micro-aggregates. This suggested that macro-aggregates were primarily responsible for the physical protection of organic carbon provided by aggregates. Organic carbon increases the capacity for carbon fixation and aggregate stability by causing small particle aggregates to bond into large particle aggregates (Wen et al., 2020). Additionally, some studies have shown that soil active carbon can bind soil particles together and promote the formation of soil aggregates (Xu and Wang, 2017). The findings of this study indicated that the soil aggregates stability was significantly positively correlated with TOPC and EOPC, but had no significant correlation with EOC/TOC. However, some study suggested that organic carbon addition affected soil aggregates stability by changing EOC/TOC, not by TOC (Wang et al., 2022).

The combined organic carbon in micro-aggregates primarily results from low-activity microbial turnover, which may impact soil nutrient availability and limit vegetation growth (Edwards and Bremner, 2010). While plant wastes are the primary source of combined organic carbon in macro-aggregates, which has a high activity and is easy for plants to absorb and use (Mizuta et al., 2015; Tian et al., 2021). Therefore, applying organic fertilizer, biochar (Islam et al., 2021), and straw (Ndzelu et al., 2020) to agricultural production might promote the formation of macro-aggregates, increase the stability of soil aggregates, and their capacity to sequester carbon increase crop productivity (Mustafa et al., 2021).

Additionally, the formation of aggregates and the fixation of organic carbon are significantly influenced by soil microorganisms (Duan et al., 2021; Han et al., 2021). Therefore, it will be necessary to expand the study on the characteristics of soil microbial changes in different aggregates better to understand the protective mechanism of aggregates on organic carbon.

## 5 Conclusion

SOC was the most important factor affecting soil aggregates stability, and it was mainly stored in macro-aggregates. Long-term application of chemical fertilizer could reduce the soil aggregates stability, storage and activity of SOC in macro-aggregates. Whereas combined application of organic fertilizer could reduce the adverse effects of chemical fertilizer, and the higher the organic fertilizer rate, the better the effect. Therefore, to increase soil nutrient availability and productivity in yellow soil, long-term application of chemical fertilizer alone should be avoided in agricultural production and replaced with a combination of chemical and organic fertilizers.

## Data availability statement

The original contributions presented in the study are included in the article/supplementary material. Further inquiries can be directed to the corresponding author.

## Author contributions

YLL designed the study and wrote the manuscript. YLL, YL, YZ, XH, YY, HZ, and TJ performed the experiments. YLL, MZ, and HX interpreted the results of the experiments and edited and revised the manuscript. YL approved the final version of the manuscript. All authors contributed to the article and approved the submitted version.
